# Physical activity is related to function and fatigue but not pain in women with fibromyalgia: baseline analyses from the Fibromyalgia Activity Study with TENS (FAST)

**DOI:** 10.1186/s13075-018-1671-3

**Published:** 2018-08-29

**Authors:** Ericka N. Merriwether, Laura A. Frey-Law, Barbara A. Rakel, Miriam B. Zimmerman, Dana L. Dailey, Carol G. T. Vance, Meenakshi Golchha, Katherine M. Geasland, Ruth Chimenti, Leslie J. Crofford, Kathleen A. Sluka

**Affiliations:** 1 Department of Physical Therapy, Steinhardt School of Culture, Education, and Human Development, New York University, New York, NY USA; 2 Department of Physical Therapy and Rehabilitation Science, University of Iowa, Iowa City, IA USA; 3 College of Nursing, University of Iowa, Iowa City, IA USA; 4 College of Public Health, University of Iowa, Iowa City, IA USA; 5 Department of Medicine/Rheumatology & Immunology, Vanderbilt University, Nashville, TN USA; 6 Department of Physical Therapy and Rehabilitation Science, 1-242 MEB, University of Iowa Carver College of Medicine, Iowa City, IA, 52422-1089 USA

**Keywords:** Pain, Fibromyalgia, PROMIS, Function, ActiGraph, Accelerometry, Fatigue, IPAQ

## Abstract

**Background:**

Although exercise is an effective treatment for fibromyalgia, the relationships between lifestyle physical activity and multiple symptomology domains of fibromyalgia are not clear. Thus, the purpose of this study was to comprehensively examine the relationships between lifestyle physical activity with multiple outcome domains in women with fibromyalgia, including pain, fatigue, function, pain-related psychological constructs, and quality of life.

**Methods:**

Women (*N* = 171), aged 20 to 70 years, diagnosed with fibromyalgia, recruited from an ongoing two-site clinical trial were included in this prespecified subgroup analysis of baseline data. Physical activity was assessed using self-report and accelerometry. Symptomology was assessed using questionnaires of perceived physical function, quality of life, fatigue, pain intensity and interference, disease impact, pain catastrophizing, and fear of movement. In addition, quantitative sensory testing of pain sensitivity and performance-based physical function were assessed. Correlation coefficients, regression analyses and between-group differences in symptomology by activity level were assessed, controlling for age and body mass index (BMI).

**Results:**

Lifestyle physical activity was most closely associated with select measures of physical function and fatigue, regardless of age and BMI. Those who performed the lowest levels of lifestyle physical activity had poorer functional outcomes and greater fatigue than those with higher physical activity participation. No relationships between lifestyle physical activity and pain, pain sensitivity, or pain-related psychological constructs were observed.

**Conclusions:**

Lifestyle physical activity is not equally related to all aspects of fibromyalgia symptomology. Lifestyle physical activity levels have the strongest correlations with function, physical quality of life, and movement fatigue in women with fibromyalgia. No relationships between lifestyle physical activity and pain, pain sensitivity, or psychological constructs were observed. These data suggest that physical activity levels are more likely to affect function and fatigue, but have negligible relationships with pain and pain-related psychological constructs, in women with fibromyalgia.

**Trial registration:**

ClinicalTrials.gov, NCT01888640. Registered on 28 June 2013.

**Electronic supplementary material:**

The online version of this article (10.1186/s13075-018-1671-3) contains supplementary material, which is available to authorized users.

## Background

The Centers for Disease Control and Prevention (CDC) recommendations exercise in for healthy adults are at least 150 minutes per week of moderate lifestyle physical activity through daily activities such as walking, stair climbing, or yard work and 2 days per week of strengthening exercises [1]. In the United States, however, the majority of the population does not meet recommended physical activity guidelines [1, 2]. Further, people with chronic pain conditions such as low back pain, osteoarthritis, and fibromyalgia (FM) often show lower levels of physical activity and greater sedentary behaviors than healthy control subjects [3–6], despite exercise being a primary treatment for many pain conditions [7]. Individuals with FM may be particularly at risk for reductions in physical activity due to pain and fatigue that often are initially exacerbated with increased activity [8]. In addition to pain and fatigue, FM is associated with reduced physical function (PF), increased pain sensitivity, reduced pain inhibition, and greater psychological comorbidities such as depression, pain catastrophizing, and fear of movement [9–14]. Thus, the multidimensional symptomology limiting quality of life (QoL) in patients with FM is due to more than just pain alone.

The many benefits of regular physical activity have been widely documented across healthy and patient populations [15]. In particular, reduced pain sensitivity has been observed in healthy adults who regularly perform vigorous daily physical activity, as evidenced by enhanced conditioned pain modulation, a measure of central pain inhibition [16, 17], and reduced temporal summation of pain [17], a measure of pain facilitation. Similarly, reductions in select measures of pain sensitivity were found in a meta-analysis of athletes versus normally active adults [18]. Higher levels of physical activity may also reduce the risk of developing chronic pain, as suggested by large epidemiological studies [19–21]. Last, in patients with chronic pain, significant improvements in pain, function, and disability occur with increased physical activity through prescribed exercise programs [7, 22, 23].

Whereas exercise is an effective treatment for those with chronic pain [7], including adults with FM [22, 23], there is limited literature examining the relationships between lifestyle physical activity and multidimensional symptomology, with conflicting results. The reported relationships between physical activity and pain outcomes in individuals with FM are variable. For example, some have found no relationship [5], an inverse relationship [6], or a positive relationship between physical activity levels and pain [24]. Also, those reporting moderate to vigorous physical activity (MVPA) levels had more pain than those with only light physical activity, but this varied with the pain scale used [24]. The rationale for these discrepant findings is unclear but may be related to methodological differences in the measurement and analysis of activity levels (e.g., self-report versus accelerometry). Further, these studies did not consider the multidimensional range of FM symptomology.

Hence, the primary purpose of this study was to comprehensively examine a variety of FM symptomology domains: pain, fatigue, PF, pain-related psychological measures, disease impact, and QoL relative to daily lifestyle physical activity (perceived and objectively assessed) in women with FM. Our primary hypothesis was that higher lifestyle physical activity levels would correspond to less pain and fatigue, reduced pain sensitivity, greater PF, lower psychological dysfunction, reduced disease impact, and better QoL. Our secondary aim was to confirm reported relationships between perceived and objectively measured physical activity in this chronic pain population.

## Methods

### Study design

The current study is a secondary analysis of baseline data from an ongoing clinical trial testing the efficacy of transcutaneous electrical nerve stimulation (TENS) in women with FM (ClinicalTrials.gov identifier NCT01888640; registered on June 28, 2013): the Fibromyalgia Activity Study with TENS (FAST). The FAST study protocol, inclusion/exclusion criteria, and procedures for this phase II, dual-site randomized controlled trial have been described previously [25], but a brief description is provided below. Further, detailed information on the validity and reliability of the multiple baseline assessments has been published previously [25, 26].

All baseline data were collected at two visits, separated by 7–10 days, in part to minimize participant burden at a single visit. Data were collected using the Research Electronic Data Capture (REDCap) system [25]. At visit 1, participants completed the consent process, were provided an accelerometer to wear on their wrist until visit 2 (*see below* for more details), and completed a demographic survey. At visit 2, participants were asked to complete a battery of instruments to evaluate self-reported pain, fatigue, physical activity, PF, QoL, disease impact, pain catastrophizing, and fear of movement. In addition, quantitative sensory testing to assess pain sensitivity and performance-based assessment of PF were performed during visit 2. Outcome data are described in detail in a paper published on the protocol for the clinical trial, including validity and reliability of the measures [25]. Below we describe those outcomes used in the current study.

### Participants

Participants from the FAST clinical trial with complete accelerometry data, recruited through October 2016, were eligible for inclusion in this planned analysis of baseline data (total recruited = 193; *N* = 171 with accelerometry). Women with FM were recruited from the communities surrounding the University of Iowa Hospitals and Clinics and Vanderbilt University Medical Center. Inclusion criteria included women between 20 and 70 years old, native English speakers, diagnosed with FM based on the 1990 American College of Rheumatology criteria [9], had a prior medical history of cervical or lumbar pain, and had stable medical management of symptoms for at least 4 weeks prior to participation. Exclusion criteria for the FAST study included pain intensity of less than 4 of 10 on the Numeric Rating Scale (NRS), unstable medical or psychiatric diagnoses, prior TENS use in the last 5 years, spinal fusion or other intervention resulting in metal in the spine (cervical, thoracic, or lumbar), pacemaker, or skin sensitivity to adhesives or products with nickel alloys. All participants provided written informed consent as approved by each local institutional review board.

Twenty-two recruited participants were ineligible for this secondary analysis due to missing accelerometry data (e.g., equipment malfunction, < 4 days wear time, or declined). Additional participants had missing data for select items on various survey assessments. Of the 171 women recruited with complete accelerometry data, some individuals had missing responses from their International Physical Activity Questionnaire (IPAQ) or other surveys; thus, total scores were not possible in up to 13 individuals (7.6%). Final sample sizes for each variable (ranging from 158 to 171) are provided in Table 1.

**Table 1:** Demographic characteristics and summary statistics (*n* = 171)

	No. of subjects	Mean (SD)	Range
Age (yr)	171	49.3 (11.5)	20–70
BMI (kg/m^2^)	171	34.4 (9.1)	19–84
FIQR (0–100)	171	56.1 (17)	17–95
Resting pain (0–10)	171	5.9 (1.5)	3–10
Resting fatigue (0–10)	171	6.4 1.7	1–10
6MWT (ft)	159	1332 (325)	300–2050
5TSTS (s)	156	14.4 (7.0)	6–53
PROMIS PF (T-score)	159	36.8 (4.9)	23.4–53.0
SF-36 PF (T-score)	160	33.1 (7.3)	19.3–55.6
SF-36 PCS (T-score)	160	32.6 (6.5)	17.3–49.5
SF-36 MCS (T-score)	160	39.9 (10.5)	15.8 – 63.6
Pain Catastrophizing Scale (0–52)	160	20.7 (13.1)	0–52
TSK (17–68)	160	36.4 (8.2)	18–58
Time spent in MVPA (min/d)	171	21.9 (23.6)	0.2–153.3
IPAQ (METs*min/wk)	158	2007 (2112)	0–9198

*Abbreviations: 5TSTS* Five Times Sit to Stand Test, *6MWT* 6-minute walk test, *BMI* Body mass index, *FIQR* Revised Fibromyalgia Impact Questionnaire, *IPAQ* International Physical Activity Questionnaire, *MAF* Multidimensional Assessment of Fatigue, *METs* Metabolic equivalent, *PCS* Physical Component Summary, *PF* Physical function, *PROMIS* Patient-Reported Outcomes Measurement Information System, *SF-36* 36-item Medical Outcomes Study Short Form Health Survey, *TSK* Tampa Scale for Kinesiophobia

### Physical activity measures

#### Self-reported physical activity

The IPAQ short form was used to measure perceived levels of physical activity over the past 7 days [27]. The IPAQ assesses time spent engaged in vigorous activities, moderate-intensity activities, walking, and sitting [27]. The survey data were processed following standardized recommendations (IPAQ guidelines, 2005), including error checking, data cleaning, and truncation at 3 hours for physical activity. Results were summarized as (1) total activity, a continuous variable reported as metabolic equivalents (METs) times minutes throughout the previous week (METs × min/week); and (2) IPAQ-defined activity categories: low, moderate, or high. High is defined as either 3 or more days of vigorous activity totaling at least 1500 METs × min/week or 7 or more days of moderate activity totaling at least 3000 METs × min/week. Moderate activity is defined as 30 or more minutes per day of moderate intensity activity for 5 days. Low activity is defined as not meeting moderate or greater activity categories. The IPAQ short form showed good repeatability (median ρ = 0.73) and good concurrent validity compared with the IPAQ long form (ρ = 0.67) [27]. The short form was chosen for its low subject burden for a clinical trial and is commonly used in clinical populations [28, 29].

#### Objectively measured physical activity

To objectively measure lifestyle physical activity, participants wore a triaxial accelerometer (ActiGraph GT1M; ActiGraph, LLC, Pensacola, FL, USA) on the nondominant wrist for 7–10 days, 24 hours per day, including showering and sleep. The study assessor provided standard visual and verbal instructions on ActiGraph use. Accelerations were collected at 30 Hz (up to ± 8 g); raw signals were extracted using ActiLife 6 software (ActiGraph, LLC). Flat-line signals prior to and following the 7–10-day period of data collection (i.e., nonwear time) were graphically evaluated and removed prior to further analyses. The raw acceleration signals were processed using a custom MATLAB program (MathWorks, Natick, MA, USA) following previously reported methodology [30]. Briefly, the resultant accelerations (g) across all three axes were determined using the root mean square (Eq. 1), called the vector magnitude. To remove the effects of gravity, 1 g was subtracted from the vector magnitude, referred to as vector magnitude − 1 (VMMO). These VMMO accelerations were averaged over each second and then across each minute, as described by Hildebrand et al. [30].

1 $$ VMMO=\sqrt{x^2+{y}^2+{z}^2}-1 $$

A modified Hildebrand approach was used to estimate activity intensity per minute (i.e., oxygen consumption [VO_2_, in ml O_2_/kg/min] for each minute throughout the 24-hour period) using a power equation (Eq. 2). The originally reported Hildebrand data, supplemented with new data collected in young and middle-aged healthy men and women, was refit to an exponential (*see* Eq. 2) equation rather than the linear equation originally reported by Hildebrand et al. [30]. The linear equation appropriately identified light or higher physical activity [30] but was unable to identify sedentary behaviors owing to the intercept being greater than standard light activity cutoffs [31]. We further validated our updated nonlinear equation with data from another laboratory involving a separate sample of healthy adults, demonstrating equal or better VO_2_ estimates for sedentary, light, walking, and vigorous activities compared with three other previously validated approaches [31]. The least accurate VO_2_ estimates occurred with moderate-intensity activities that were chosen specifically for isolated arm or leg movements (cycling, typing while walking, throwing), as expected for wrist accelerometry [31].

2 $$ {VO}_2=0.901\ast {\left( VMMO\ in\ milli\ {g}^{\prime }s\right)}^{0.534} $$

Daily minutes of MVPA were calculated as the average number of minutes per 24-hour interval spent in moderate or vigorous activity, operationally defined as VO_2_ intensities greater than 11 ml O_2_/kg/min [31]. Approximate tertiles of accelerometry average daily MVPA were used to define three physical activity groups in this patient population: very low (0–9 minutes MVPA/day), low (10–21 minutes MVPA/day), and moderate (> 21 minutes MVPA/day). In addition to dividing the cohort into three approximately equal samples, an average of 21 minutes per day equates to 150 minutes per week, which is consistent with CDC physical activity guidelines. However, whereas this meets the CDC guidelines, which do not mention minimum bout durations, it may not meet American College of Sports Medicine guidelines, which indicate MVPA should occur in bouts of 10 or more minutes. No adjustments for bout duration were made. Thus, for both the self-reported and objectively measured assessments of physical activity, both continuous and categorical variables were assessed and analyzed.

### Pain and pain sensitivity assessments

#### Current resting and movement pain intensity

Pain intensity at rest (resting pain) and during movement (movement pain) were assessed using the verbal 0–10 NRS at both baseline visits and averaged. Movement pain was assessed at 5 minutes during the 6-minute walk test (6MWT) and immediately following the Five Times Sit to Stand Test (5TSTS). The 11-point NRS pain scale was anchored with 0 = “no pain” and 10 = “worst pain imaginable.”

#### Overall pain severity and interference

The Brief Pain Inventory (BPI) short form was used to measure overall pain severity and pain interference. This 15-item instrument queries pain intensity (pain severity) and the impact of pain on daily function (pain interference) [32]. The BPI pain severity scale assesses pain intensity at its “worst,” “least,” “average,” and “right now.” Pain severity items are assessed on a 0–10 scale, anchored with 0 = “no pain” and 10 = “pain as bad as you can imagine.” Pain interference items assess seven domains of daily activities (general activity, walking, work, mood, enjoyment of life, relations with others, sleep) a 0–10 scale anchored with 0 = “does not interfere” to 10 = “completely interferes” [32, 33].

#### Deep tissue mechanical pain threshold

Pressure pain threshold (PPT), a measure of deep tissue pain thresholds, was assessed at three sites (cervical, lumbar, anterior lower leg) using a digital pressure algometer at a rate of 40 kPa/second using a 1-cm^2^ round tip (Somedic AB, Sösdala, Sweden). These sites included two spine locations, because these are frequently painful regions with FM, and a peripheral site, the lower leg, which is commonly assessed in other research studies including in FM [8]. Participants were instructed to press and release a button when the pressure sensation first transitioned to pain (NRS ~ 1). Following two practice tests on the forearm, four repetitions were performed at each of the cervical and lumbar spine sites (two on each side of the spinous processes) and three repetitions at the lower leg. The mean value at each site was used for analyses. Lower PPTs indicate greater pain sensitivity.

#### Pain inhibition

Conditioned pain modulation (CPM) is an assessment of descending inhibitory pain processing (the “pain inhibits pain” effect) and was measured using PPTs as the test stimuli before and after cold water immersion as the painful conditioning stimulus [8, 34–36]. PPTs were measured at the lumbar and right lower leg before and immediately after immersion of the participant’s left foot in 4 °C water just proximal to the lateral malleolus [8, 34]. An increase in PPT (less pain sensitivity) after the conditioning stimulus (ratio of post-/pre-PPT values > 1) demonstrates presence of descending inhibition, whereas no change or a reduction in PPT (ratio ≤ 1) demonstrates the absence of descending inhibition.

### Fatigue assessment

#### Current resting and movement fatigue intensity

Fatigue intensity at rest (resting fatigue) and during movement (movement fatigue) were assessed using the verbal 0–10 NRS at both baseline visits and averaged. Movement fatigue was assessed at 5 minutes during the 6MWT and immediately following the 5TSTS. The 11-point NRS-Fatigue scale is anchored with 0 = “no fatigue” and 10 = “worst fatigue imaginable” as we previously published [37].

#### Overall multidimensional fatigue

The Multidimensional Assessment of Fatigue (MAF) survey assesses perception of fatigue across multiple domains and is commonly used in rheumatologic populations [38, 39]. The MAF features 16 items with 4 domains that include distress, severity, timing of fatigue onset, and impact on activities of daily living. Composite scores from each domain comprise the Global Fatigue Index (GFI), with anchors of 0 = “no fatigue” to 50 = “extreme fatigue.” Higher scores indicate greater fatigue and fatigue impact.

### Physical function assessments

#### Self-reported physical function

The Patient-Reported Outcomes Measurement Information System–Physical Function 10a static short-form (PROMIS-PF) [26] was used to assess PF. Responses are transformed to standardized T-scores using the PROMIS conversion table, with 50 representing the general population mean and SD of 10. Higher scores indicate better perceived function. The PF subscale from the 36-item Medical Outcomes Study Short Form Health Survey (SF-36) [40] was also assessed as a secondary measure of PF (SF-36 PF) [26]. Participants completed the SF-36 (*see below*), and the raw response data for the ten items representing PF were converted to a standardized T-score, with higher scores indicating better overall function. We have previously validated this form in FM [26].

#### Physical endurance

The 6MWT was used to measure physical endurance. The 6MWT measures the distance participants walk on a 100-foot walkway in 6 minutes, with rest periods allowed if necessary [41]. The 6MWT is frequently used in clinical practice and has been used previously to measure physical performance in individuals with FM [8].

#### Lower extremity strength

The 5TSTS was used as a measure of lower extremity strength [42]. Participants stand up from and sit down in a chair with standard seat height five times as quickly as possible. The time in seconds required to complete the test is recorded.

### Pain-related psychological assessments

#### Pain catastrophizing

Pain catastrophizing was measured using the Pain Catastrophizing Scale. The Pain Catastrophizing Scale is a 13-item instrument that measures the extent to which individuals experience different catastrophic thoughts and feelings related to pain, scored from 0 = “not at all” to 4 = “always” [43]. Scores can range from 0 to 52, with higher scores reflecting greater pain catastrophizing.

#### Fear of movement

Fear of movement was measured using the Tampa Scale for Kinesiophobia (TSK). The TSK is a 17-item Likert scale instrument that evaluates fear of movement and reinjury associated with a variety of physical activities. TSK total score is the sum of the all items, with scores ranging from 17 to 68. Higher scores indicate greater fear of movement or reinjury [44].

### Disease impact and quality-of-life assessments

#### Disease impact

Disease impact was measured using the Revised Fibromyalgia Impact Questionnaire (FIQR) at both baseline visits. The FIQR contains 21 items divided into 3 domains: (1) “function” (9 items), (2) “overall impact” (2 items), and (3) “symptoms” (10 items) [45]. The total score of the FIQR is the sum of the weighted domain scores and was averaged across both visits. Higher scores indicate greater disease severity and impact.

#### Quality of life

The SF-36 was used to measure QoL. This 36-item instrument is a common QoL assessment tool [40]. Physical and emotional QoL domains were assessed using the Physical Component Summary (PCS) and the Mental Component Summary scores, respectively, where higher T-scores reflect better QoL.

### Statistical analyses

Descriptive statistics were generated for all variables (mean, SD in text and tables; mean, SE in figures). Normality assumptions of the variables were assessed using the Kolmogorov-Smirnoff test. When necessary, natural logarithm transformations were performed to meet normality assumptions, but are reported in original units for clarity. Pearson’s correlation coefficients were assessed between measures within each FM outcome domain: pain, pain sensitivity, fatigue, self-reported and performance-based function, psychological traits, and QoL and between the two continuous physical activity variables (accelerometry min of MVPA and IPAQ total METs × min/wk) to characterize relationships between related variables.

To fully characterize the relationships between lifestyle physical activity and the multiple FM symptomology domains, two statistical approaches were used. First, Pearson’s correlation coefficients (95% CI) were computed between lifestyle physical activity, both accelerometry and self-reported measures, and the multiple assessments of each symptomology construct. These correlations were assessed with and without adjustment for age and body mass index (BMI) using partial and bivariate correlations, respectively, because obesity (BMI > 30 kg/m^2^) and age (> 60 years) often result in lower levels of lifestyle physical activity [46, 47]. The second approach evaluated for differences in the symptomology variables across categorical levels of physical activity (IPAQ and accelerometry MVPA) using analysis of variance (ANOVA), with and without adjustments for age and BMI. Post hoc group differences were assessed using Tukey’s test as needed. All statistical analyses were performed using SAS version 9.4 statistical software (SAS Institute Inc., Cary, NC, USA) or IBM SPSS Statistics version 24.0 software (IBM, Armonk, NY, USA). Significance was set at *p* ≤ 0.05 for Kolmogorov-Smirnoff tests, demographic comparisons, and necessary post hoc tests. Due to the multiple correlations and ANOVAs evaluating each FM outcome measure, significance was set at *p* ≤ 0.01 for all tests to minimize the likelihood of type I errors while not overly inflating the likelihood of a type II error.

## Results

Demographic characteristics and summary statistics for all physical activity and multidimensional FM symptomology variables are presented in Table 1. Participants had an average (SD) age of 49.4 (11.5) years and BMI of 34.4 (9.1) kg/m^2^. Most participants had been diagnosed with FM for less than 10 years (58%) and were white/Caucasian (94%). Physical activity continuous variables (accelerometry and self-report) are presented with original units for clarity in Table 1 but were transformed using a natural logarithm to meet normality assumptions before statistical analyses.

Self-report (IPAQ) and objective (MVPA) assessments of lifestyle physical activity had a moderate positive correlation (*r* = 0.32, *p* < 0.001). Higher levels of MVPA, accelerometry-based MVPA, was associated with younger age (*p* < 0.001) and lower BMI (*p* < 0.001), with age and BMI explaining from 6–12% of the variance in accelerometry physical activity. Using self-report, however, this relationship was weaker for BMI (4% variance explained; *p* < 0.02) and not significant for age. These results are provided in Additional file 1: Table S1.

### Correlations between physical activity and FM symptomology

Correlational analyses revealed limited relationships between objective (Table 2) or self-report (Table 3) measures of physical activity and measures representing the multiple symptomology domains of FM, contrary to our initial hypotheses. Greater MVPA (Table 2) was significantly related to higher perceived (PROMIS PF, SF-36 PF) and performance-based (6MWT, 5TSTS) PF, less movement fatigue during the 6MWT, and greater physical QoL (SF-36 PCS) in women with FM. These relationships were maintained for four of the six significant relationships after adjusting for age and BMI. However, MVPA was not related to pain, pain sensitivity, pain-related psychological constructs, disease impact, or emotional QoL (Table 2).

**Table 2:** Correlations between objective physical activity and fibromyalgia symptomology assessments

Outcome domain	Outcome variable	No. of subjects	Pearson’s correlation (unadjusted)			Partial correlation (BMI and age-adjusted)		
			*r*	(95% CI)	*p* Value	*r*	(95% CI)	*p* Value
**Performance-Based Function**	**Endurance (6MWT, ft)**	159	**0.43**	(0.30, 0.55)	**< 0.001**	**0.28**	(0.12, 0.41)	**< 0.001**
	**Strength (5TSTS time, s) (ln)**	156	**− 0.17**	(− 0.32, − 0.01)	**0.01**	− 0.02	(− 0.18, 0.14)	0.81
**Self-reported function**	**PROMIS PF**	159	**0.25**	(0.09, 0.39)	**0.002**	0.15	(− 0.01, 0.30)	0.07
	**SF-36 PF**	160	**0.31**	(0.15, 0.43)	**< 0.001**	**0.20**	**(0.06, 0.38)**	**0.01**
Pain	Resting pain (0–10)	171	− 0.04	(− 0.19, 0.11)	0.61	0.02	(− 0.14, 0.18)	0.80
	Movement pain (6MWT)	160	− 0.09	(− 0.24, 0.07)	0.28	− 0.01	(− 0.17, 0.14)	0.87
	Movement pain (5TSTS)	159	− 0.04	(− 0.19, 0.12)	0.65	− 0.00	(− 0.16, 0.16)	0.98
	BPI severity	160	0.00	(− 0.16, 0.16)	0.99	0.01	(− 0.14, 0.17)	0.87
	BPI interference	160	− 0.05	(− 0.20, 0.11)	0.52	− 0.02	(− 0.17, 0.14)	0.84
Pain sensitivity	PPT cervical (ln)	159	− 0.10	(− 0.25, 0.06)	0.21	− 0.08	(− 0.24, 0.08)	0.31
	PPT lumbar (ln)	159	− 0.04	(− 0.19, 0.12)	0.65	− 0.03	(− 0.19, 0.13)	0.70
	PPT leg (ln)	159	0.01	(− 0.16, 0.15)	0.92	0.01	(− 0.16, 0.15)	0.88
	CPM lumbar (%)	138	0.14	(− 0.03, 0.30)	0.10	0.14	(− 0.04, 0.30)	0.12
	CPM leg (%)	134	0.07	(− 0.11, 0.23)	0.45	0.11	(− 0.06, 0.28)	0.21
**Fatigue**	Resting fatigue (0–10)	171	− 0.11	(− 0.25, 0.05)	0.17	− 0.01	(− 0.17, 0.14)	0.86
	**Movement fatigue (6MWT)**	160	**− 0.20**	(− 0.35, − 0.05)	**0.01**	**− 0.20**	(− 0.35, −0.05)	**0.01**
	Movement fatigue (5TSTS)	160	0.00	(− 0.15, 0.16)	0.96	− 0.02	(− 0.18, 0.14)	0.81
	Multidimensional (MAF)	159	− 0.05	(− 0.21, 0.11)	0.52	− 0.05	(− 0.20, 0.11)	0.58
Psychological constructs	Pain Catastrophizing (Pain Catastrophizing Scale)	160	0.05	(− 0.10, 0.21)	0.52	0.01	(− 0.15, 0.16)	0.93
	Fear of movement (TSK)	160	− 0.01	(− 0.17, 0.14)	0.89	− 0.01	(− 0.17, 0.15)	0.89
Disease impact	FIQR total	171	− 0.12	(− 0.27, 0.03)	0.12	− 0.10	(− 0.25, 0.06)	0.24
**Quality of life**	**Physical (SF-36 PCS)**	158	**0.27**	(0.11, 0.41)	**0.001**	**0.22**	(0.06, 0.37)	**0.006**
	Emotional (SF-36 MCS)	158	− 0.01	(− 0.17, 0.14)	0.89	− 0.01	(− 0.17, 0.16)	0.99

*Abbreviations: MVPA* Moderate to vigorous activity, *6MWT* 6-minute walk test, *5TSTS* Five Times Sit to Stand Test, *PF* Physical Function T-score for Patient-Reported Outcomes Measurement Information System physical function static short form, *SF-36 PCS* 36-item Medical Outcomes Study Short Form Health Survey Physical Component Summary, *BPI* Brief Pain Inventory, *PPT* Pressure pain threshold, *CPM* Conditioned pain modulation, *MAF* Multidimensional Assessment of Fatigue, *TSK* Tampa Scale for Kinesiophobia, *FIQR* Revised Fibromyalgia Impact Questionnaire, *SF-36 MCS* 36-item Medical Outcomes Study Short Form Health Survey Mental Component Score

(ln) The natural logarithm of accelerometry MVPA was used

All significance are bolded

**Table 3:** Correlations between self-reported physical activity (International Physical Activity Questionnaire total) and fibromyalgia symptomology assessments

Outcome domain	Outcome variable	No. of subjects	Pearson’s correlation (unadjusted)			Partial correlation (BMI and age-adjusted)		
			*r*	95% CI	*p* Value	*r*	95% CI	*p* Value
**Performance-Based Function**	**Endurance (6MWT, ft)**	157	**0.30**	(0.12, 0.40)	**< 0.001**	**0.23**	(0.05, 0.30)	**0.005**
	Strength (5TSTS time, s) (ln)	154	− 0.09	(− 0.21, 0.11)	0.26	− 0.02	(− 0.13, 0.18)	0.86
**Self-reported function**	**PROMIS PF**	157	**0.27**	(0.10, 0.39)	**0.001**	**0.21**	(0.07, 0.34)	**0.009**
	**SF-36 PF**	157	**0.21**	(0.07, 0.35)	**0.009**	0.15	(− 0.01, 0.32)	0.07
Pain	Resting pain (0–10)	158	0.01	(− 0.15, 0.16)	0.95	− 0.03	(− 0.12, 0.19)	0.73
	Movement pain (6MWT)	158	0.02	(− 0.13, 0.18)	0.77	0.03	(− 0.09, 0.22)	0.70
	Movement pain (5TSTS)	157	− 0.01	(− 0.17, 0.15)	0.88	− 0.02	(− 0.15, 0.17)	0.85
	BPI severity	158	− 0.12	(− 0.24, 0.07)	0.15	− 0.11	(− 0.23, 0.08)	0.17
	BPI interference	158	− 0.15	(− 0.24, 0.04)	0.06	− 0.13	(− 0.22, 0.04)	0.12
Pain sensitivity	PPT cervical (ln)	157	0.15	(− 0.02, 0.29)	0.06	0.17	(0.01, 0.32)	0.04
	PPT lumbar (ln)	157	0.16	(0.02, 0.30)	0.04	0.17	(0.01, 0.32)	0.04
	PPT leg (ln)	157	0.11	(− 0.06, 0.29)	0.18	0.12	(− 0.02, 0.26)	0.13
	CPM lumbar (%)	136	0.06	(− 0.11, 0.23)	0.46	0.05	(− 0.13, 0.21)	0.54
	CPM leg (%)	132	− 0.03	(− 0.22, 0.15)	0.76	− 0.01	(− 0.20, 0.17)	0.94
Fatigue	Resting fatigue (0–10)	158	− 0.08	(− 0.18, 0.03)	0.34	− 0.03	(− 0.18, 0.16)	0.69
	Movement fatigue (6MWT)	158	− 0.08	(− 0.18, 0.03)	0.30	− 0.06	(− 0.16, 0.15)	0.45
	Movement fatigue (5TSTS)	158	− 0.04	(− 0.18, 0.14)	0.47	− 0.04	(− 0.17, 0.14)	0.64
	Multidimensional (MAF)	157	− 0.02	(− 0.18, 0.14)	0.79	− 0.01	(− 0.17, 0.15)	0.99
Psychological constructs	Catastrophizing (Pain Catastrophizing Scale)	158	0.01	(− 0.21, 0.24)	0.94	− 0.02	(− 0.10, 0.22)	0.80
	Fear of movement (TSK)	158	− 0.17	(− 0.31, − 0.02)	0.03	− 0.16	(− 0.29, −0.02)	0.04
Disease impact	FIQR total	158	− 0.02	(− 0.17, 0.14)	0.83	− 0.07	(− 0.15, 0.16)	0.40
**Quality of life**	**Physical (SF-36 PCS)**	156	**0.22**	(0.06, 0.35)	**0.006**	0.18	(0.02, 0.32)	0.03
	Emotional (SF-36 MCS)	156	− 0.06	(− 0.21, 0.09)	0.48	− 0.05	(− 0.18, 0.08)	0.55

*Abbreviations: MVPA* Moderate to vigorous activity, *6MWT* 6-minute walk test, *5TSTS* Five Times Sit to Stand Test, *PF* Physical Function T-score for Patient-Reported Outcomes Measurement Information System physical function static short form, *SF-36 PCS* 36-item Medical Outcomes Study Short Form Health Survey Physical Component Summary, *BPI* Brief Pain Inventory, *PPT* Pressure pain threshold, *CPM* Conditioned pain modulation, *MAF* Multidimensional Assessment of Fatigue, *TSK* Tampa Scale for Kinesiophobia, *FIQR* Revised Fibromyalgia Impact Questionnaire, *SF-36 MCS* 36-item Medical Outcomes Study Short Form Health Survey Mental Component Score

(ln) The natural logarithm of accelerometry IPAQ total was used

All significance are bolded

Similar positive correlations were observed for self-reported physical activity with perceived and performance-based PF as well as physical QoL (Table 3). Adjustment for age and BMI resulted in only minor changes to correlation coefficient estimates (Table 3). Similar to objective correlations above, no significant relationships between perceived activity and pain, pain sensitivity, psychological constructs, or disease impact reached significance (*p* < 0.01). However, the relationship between self-reported physical activity and PPTs in the cervical and lumbar spine regions and fear of movement nearly reached significance (*p* < 0.05).

### Differences by activity classification

Several demographic characteristics varied between activity group classifications based on MVPA but not self-report assessment (Table 4). Women in the lowest accelerometry MVPA tertile (very low; 0–9 min/d MVPA) were older and had higher BMI than those achieving moderate MVPA levels (moderate; > 21 min/d MVPA). However, no significant differences in age or BMI were noted between any self-report (IPAQ)-based activity classifications (Table 4).

**Table 4:** Summary statistics (mean, SD) of age and body mass index between activity classifications

	Accelerometry Activity classification				IPAQ Activity classification			
	Very low (*n* = 58)	Low (*n* = 56)	Moderate (*n* = 57)	p Value	Low (*n* = 73)	Moderate (*n* = 43)	High (*n* = 42)	p Value
Age (years)	54.4^a^ (10.2)	48.5^b^ (10.9)	45.0^b^ (11.46)	**< 0.0001**	50.7 (11.2)	49.3 (11.8)	46.6 (12.2)	0.194
BMI (kg/m^2^)	37.4^a^ (10.1)	35.0^a^ (8.7)	30.6^b^ (7.0)	**< 0.0001**	35.9 (10.2)	33.3 (7.4)	32.9 (9.3)	0.166

Superscript letters denote which groups differed from one another, where matching superscripts indicate no significant difference

All significance are bolded

**Fig. 1:**
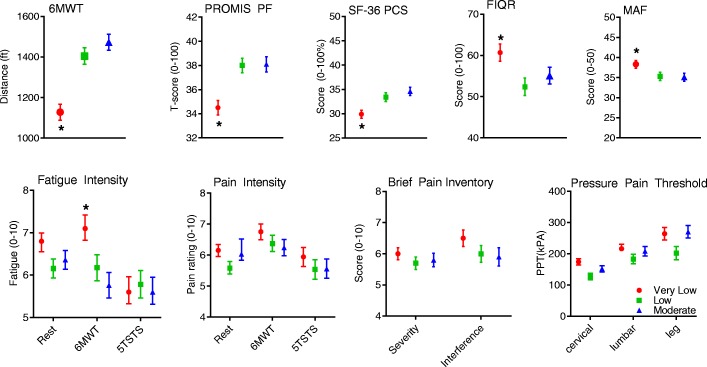
Mean (SEM) fibromyalgia symptomology by accelerometry activity classification (min/d of moderate to vigorous activity). Select function, fatigue, quality of life, and pain measures are shown. Those with the lowest levels of objectively measured physical activity had the lowest function, highest fatigue with walking, and lowest physical quality of life, but no differences in pain, pain sensitivity or disease impact were noted. * *p* < 0.01. *5TSTS* Five Times Sit to Stand Test, *6MWT* 6-minute walk test, *FIQR* Revised Fibromyalgia Impact Questionnaire, *MAF* Multidimensional Assessment of Fatigue, *PCS* Physical Component Summary, *PF* Physical function, *PROMIS* Patient-Reported Outcomes Measurement Information System, *SF-36* 36-item Medical Outcomes Study Short Form Health Survey

When comparing FM symptomology domains by activity category for MVPA (Fig. 1), there were significant between group differences in function-related indices (*p* ≤ 0.01). Post hoc analyses revealed that those achieving the very lowest activity levels (< 9 min/d MVPA) had shorter 6MWT distance, lower self-reported function (PROMIS and SF-36 PF scales, only PROMIS shown Fig. 1, lower movement fatigue during the 6MWT, and lower physical QoL (SF 36 PCS) compared with those with low to moderate minutes of MVPA (10+ min/d MVPA). No differences between accelerometry groups were noted for pain, pain sensitivity, resting or global fatigue, or disease impact. Adjustment of age and BMI did not alter the findings. (*See* Additional file 1: Table S2 for full summary of *p* values.)

**Fig. 2:**
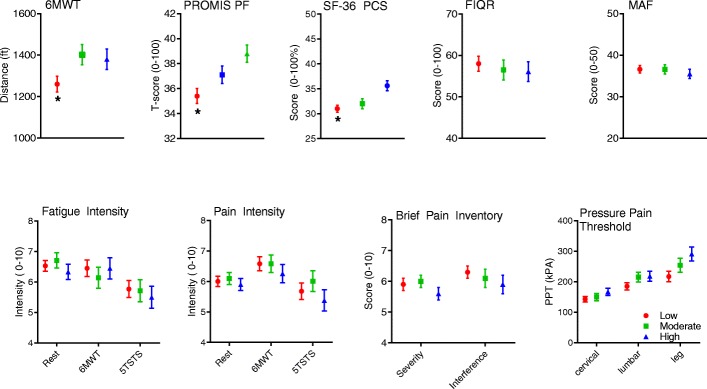
Mean (SEM) fibromyalgia symptomology by self-reported International Physical Activity Questionnaire short form activity classification. Select function, fatigue, quality of life, and pain measures are shown. Those with the lowest levels of self-reported physical activity had the lowest function and greater fatigue. * *p* < 0.01. *5TSTS* Five Times Sit to Stand Test, *6MWT* 6-minute walk test, *FIQR* Revised Fibromyalgia Impact Questionnaire, *MAF* Multidimensional Assessment of Fatigue, *PCS* Physical Component Summary, *PF* Physical function, *PROMIS* Patient-Reported Outcomes Measurement Information System, *SF-36* 36-item Medical Outcomes Study Short Form Health Survey

Similarly, when comparing across self-report activity categories (Fig. 2), only the perceived function (PROMIS and SF-36 PF), and physical QoL (SF-36 PCS) differed across groups, with or without adjusting for age and BMI. Post hoc tests revealed significant differences between the low and high activity groups, again where higher function and physical QoL were seen only in the highest self-reported activity group. The only discrepancies between categorical and correlational analyses were the lack of difference in 6MWT distance and associated fatigue ratings across IPAQ categories; yet, a significant correlation between 6MWT and total IPAQ activity was seen (*see* Additional file 1: Table S2 for all *p* values). Correlations between each assessment within each symptomology domain are provided in Additional file 1: Tables S3–S7.

## Discussion

The primary finding of this study is that lifestyle physical activity is most closely associated with function, physical QoL, and movement fatigue in women with FM. These findings were consistently observed for self-reported and objectively measured assessments of daily lifestyle physical activity. Accordingly, those with the lowest levels of lifestyle physical activity have worse function, lower physical QoL, and more fatigue with movement. However, contrary to our initial hypotheses, there were no significant associations between lifestyle physical activity and the pain, pain sensitivity, pain-related psychological constructs, resting fatigue, emotional QoL, or disease impact measures.

Our findings are consistent with several previous studies in patient populations but differ somewhat from healthy adults. In one study of patients with FM, there were no observed relationships between peak levels of physical activity or steps per day, and general pain ratings were observed in one study [6]. The al-Andalus study found no association between MVPA and pain using the FIQR pain scale and resting pain ratings, nor with PPTs, a measure of pain sensitivity in those with FM [24, 48]. However, the al-Andalus study researchers observed a significant but weak association between MVPA and pain using the SF-36 pain subscale [24]. It is not easily explained why this one pain scale but not the others would be related to MVPA. In healthy adults without baseline elevated pain, it is not feasible to study the associations between activity and pain. However, epidemiological data demonstrate a reduced incidence of chronic pain in moderately active individuals compared with sedentary individuals [20, 49]. We also found no relationship between pain sensitivity (PPTs) or pain inhibition (CPM) and daily physical activity in FM, which differs from findings in healthy control subjects, who often exhibit reduced pain sensitivity and/or greater pain inhibition with greater physical activity [17, 50].

People with FM often exhibit altered central nervous system pain processing. These alterations include less descending control of pain inhibition (CPM) and/or greater pressure pain sensitivity, thought to represent a heightened state of central sensitization [21]. Thus, it is possible that central sensitization in a chronic widespread pain condition may be less influenced by physical activity than when in a healthy state. Alternatively, physical activity levels in this specific FM cohort may be subthreshold to influence pain sensitivity.

In support, a randomized controlled trial designed to increase steps per day in people with FM found reduced pain after 12 weeks compared with an education-only intervention [51]. These differences were lost at 6 and 12 months, when daily steps declined toward preintervention levels, supporting the hypothesis that there is a minimal dose of physical activity needed to modify pain outcomes.

This inconsistency between cross-sectional observations and interventional trials may be a result of underlying pathophysiological pain mechanisms or human behavior. Indeed, we have previously shown in animal studies that acute increases in physical activity can exacerbate pain, whereas regular physical activity over time plays a protective role in reducing or preventing chronic pain [49, 52–55]. Thus, whether activity is routine or recently changed may influence its relationship with pain. Further, individuals may titrate their daily activity levels to their pain so that they are more active when their pain is lower and less active when experiencing greater pain. Prior studies support this premise, where accelerometry-measured physical activity levels were lower when individuals with FM reported higher pain [6], and women with FM are less active than their age-matched control subjects [4, 24]. This inverse relationship, combined with any possible positive relationship between lifestyle physical activity and pain, could partially explain the lack of association between monitored lifestyle activity and pain outcomes. Conversely, lifestyle activity is also dependent on choice, and some choose to be active or inactive regardless of pain levels. The information in the current study may assist clinicians in discussing the benefits and use of exercise as a pain-relieving and general health promotion strategy with patients. Specifically, it is advisable that health care providers acknowledge that exercise and increasing daily activity are likely to improve function and fatigue but may not reduce pain.

The significant relationships between daily physical activity and function suggest a clear connection between lifestyle daily activity and muscle performance (e.g., strength, endurance) even in the FM patient population. Physical activity guidelines, based largely on reducing cardiovascular disease risk, suggest a minimum of 30 min/d of moderate physical activity [1]. However, whether lower levels of daily physical activity are beneficial for chronic pain patients has not been well defined [56]. The current study shows, for the first time to our knowledge, that just 10 min/d or more of MVPA is associated with better perceived function, 6MWT performance, and physical QoL, regardless of age and BMI. No further benefit in function was observed in those averaging 21+ min/d of MVPA. Indeed, CDC physical activity guidelines and other investigators concur that some physical activity is better than none [1, 57–59]. It remains unclear if further increases in MVPA would result in greater influences on pain, fatigue, or other FM symptom domains, because few in our patient population were vigorously active.

Our results showing an association with lifestyle physical activity and movement fatigue, but not overall perceptions of fatigue, suggest that movement-evoked fatigue is a unique construct that could be modulated by physical activity and exercise. In support of this, prior work shows that individuals with FM with higher MVPA report less fatigue than those with lower MVPA [24], and increasing activity through exercise reduces fatigue in a variety of conditions associated with fatigue, including pain [23, 60, 61]. However, in those with FM, increasing physical activity levels by 50% over 12 weeks had no effect on perceived fatigue measured using the Fatigue Severity Scale [51].

Increasing physical activity and exercise is a first-line nonpharmacological treatment for FM, and systematic reviews show reductions in pain and depression, as well as improved global health and PF [22, 23, 62, 63]. However, systematic reviews report varied effect sizes across FM outcomes and exercise types [62–64]. For example, aerobic exercise produced no to small effect sizes on pain; small effect sizes on fatigue, depression, and global health; and medium effect sizes on PF [63, 64]. This larger effect size of increases in physical activity on function than on the other symptom domains is consistent with our current findings with lifestyle physical activity. Accordingly, we propose that the first expectation for improvement due to increased physical activity in the FM population should be functional gains and improved physical QoL, with secondary potential for improvements in other domains.

Measuring physical activity is inherently challenging, regardless of the method of measurement. Although both objective and subjective methods intend to assess the same construct, lifestyle physical activity, it is well documented that correlations between survey-based and accelerometry-based activity assessment range between *r* = 0.14 and *r* = 0.56 [65]. A major limitation of self-report activity assessments is recall bias, which has led to the belief that accelerometry is a better assessment of lifestyle physical activity. However, there are a number of noted limitations for accelerometry measurements, including the ability to measure only dynamic physical activity, dependence on accelerometer wear placement (e.g., wrist vs. hip), and sensitivity to the analysis used (e.g., step count, activity counts, transformation equations of the raw g signals) [34]. In the current study, both methods resulted in consistent findings with associations between greater physical activity and better function, physical QoL, and movement fatigue, as well as the lack of relationship between physical activity and pain, pain-related psychological constructs, and mental QoL, suggesting the findings are robust. Thus, for this population, either measurement approach is useful, particularly because the relatively low burden of the IPAQ short form makes it a feasible assessment in clinic settings.

The current study has several limitations. First, this is a cross-sectional study and as such we cannot determine causation. Accordingly, better function and QoL may result in greater physical activity levels as opposed to greater physical activity resulting in improved function and QoL. Further, activity over a 7-day period may not be representative of habitual physical activity levels. However, longer durations are not feasible for accelerometry and are likely to involve greater recall bias errors for self-report. Finally, participants in the parent clinical trial, used for these analyses, included only women with NRS pain ratings of at least 4 of 10 and thus may not represent all FM populations, such as those with milder symptoms or men.

## Conclusions

Lifestyle physical activity levels have the strongest correlations with function, physical QoL, and movement fatigue in women with FM. These relationships occurred with low levels of moderate physical activity (> 10 min/d), well below most physical activity guidelines. No relationships between lifestyle physical activity and pain, pain sensitivity, or psychological constructs were observed, indicating that some individuals with high pain, pain catastrophizing, or fear remain active, whereas others do not. Clinically, these data support that increasing daily physical activity has the potential to improve function, improve physical QoL, and reduce movement-evoked fatigue in this population.

## Additional file

Additional file 1: **Table S1.** Correlation coefficients between objective and self-report measures of physical activity, age, and BMI. **Table S2.** *p* values of between-group differences in FM symptomology by objective and self-report activity classifications. **Table S3.** Correlation coefficients between function variables. **Table S4.** Correlation coefficients between pain variables. **Table S5.** Correlation coefficients between pain sensitivity variables. **Table S6.** Correlation coefficients between fatigue variables. **Table S7.** Correlation coefficients between psychological constructs, disease impact, and QoL variables. (DOCX 20 kb)

## Abbreviations

5TSTS: Five Times Sit to Stand Test
6MWT: Six minute walk test
ANOVA: Analysis of variance
BMI: Body mass index
BPI: Brief Pain Inventory
CDC: Centers for Disease Control and Prevention
CPM: Conditioned pain modulation
FAST: Fibromyalgia Activity Study with TENS
FIQR: Revised Fibromyalgia Impact Questionnaire
FM: Fibromyalgia
GFI: Global Fatigue Index
IPAQ: International Physical Activity Questionnaire
MAF: Multidimensional Assessment of Fatigue
MCS: Mental Component Summary
METs: Metabolic equivalent
MVPA: Moderate to vigorous activity
NRS: Numeric Rating Scale
PCS: Physical Component Summary
PF: Physical function
PPT: Pressure pain threshold
PROMIS: Patient-Reported Outcomes Measurement Information System
QoL: Quality of life
REDCap: Research Electronic Data Capture
SF-36: 36-item Medical Outcomes Study Short Form Health Survey
TENS: Transcutaneous electrical nerve stimulation
TSK: Tampa Scale for Kinesiophobia
VMMO: Vector magnitude − 1
