# Supplementary material for: Physical activity is related to function and fatigue but not pain in women with fibromyalgia: baseline analyses from the Fibromyalgia Activity Study with TENS (FAST)
Source: Arthritis Res Ther. 2018 Aug 29;20:199. doi: 10.1186/s13075-018-1671-3 (PMC6116369; doi:10.1186/s13075-018-1671-3)
Supplement: Supplementary file 1 — Table S1. Correlation coefficients between objective and self-report measures of physical activity, age, and BMI. Table S2. p values of between-group differences in FM symptomology by objective and self-report activity classifications. Table S3. Correlation coefficients between function variables. Table S4. Correlation coefficients between pain variables. Table S5. Correlation coefficients between pain sensitivity variables. Table S6. Correlation coefficients between fatigue variables. Table S7. Correlation coefficients between psychological constructs, disease impact, and QoL variables. (DOCX 20 kb) [file 13075_2018_1671_MOESM1_ESM.docx]

**Table S1. Correlation Coefficients between objective and self-report measures of physical activity, age, and BMI.**

|  | MVPA  (min) | Age  (yrs) | BMI  (kg/m^2^) |
| --- | --- | --- | --- |
| Accelerometry MVPA (min) | - | **-0.34**** | **-0.25**** |
| IPAQ Total (MET*min/week) | **0.32**** | -0.12 | -0.19 |

Note: natural log transformations were applied to all physical activity variables.

* p ≤ 0.01; ** P ≤ 0.001

**Table S2. P-values of between-group differences in FM Symptomology by objective and self-report activity classifications.**

| **Outcome Domain** | **Outcome variable** | **n** | **MVPA Classification** | | **IPAQ Classification** | |
| --- | --- | --- | --- | --- | --- | --- |
|  |  |  | **P-value (unadj.)** | **P-value (adj BMI, age)** | **P-value (unadj.)** | **P-value (adj BMI, age)** |
| Function | **Endurance (6MWT, ft)** | 159 | **< 0.001** | **< 0.001** | 0.04 | 0.18 |
|  | Strength (5TSTS time, s) (ln) | 156 | 0.19 | 0.57 | 0.26 | 0.30 |
| Self-reported function | **PROMIS - PF** | 159 | **< 0.001** | **0.003** | **0.001** | **0.009** |
|  | **SF-36 PF** | 160 | **< 0.001** | **0.005** | **0.001** | **0.007** |
| Pain | Resting Pain (NRS) | 171 | 0.11 | 0.10 | 0.89 | 0.86 |
|  | Movement Pain (6MWT) | 160 | 0.36 | 0.86 | 0.71 | 0.79 |
|  | Movement Pain (5TSTS) | 159 | 0.59 | 0.77 | 0.44 | 0.41 |
|  | BPI Severity | 160 | 0.52 | 0.55 | 0.54 | 0.57 |
|  | BPI Interference | 160 | 0.22 | 0.43 | 0.62 | 0.76 |
| Pain sensitivity† | PPT Cervical (ln) | 159 | 0.03 | 0.04 | 0.10 | 0.07 |
|  | PPT Lumbar (ln) | 159 |  |  |  |  |
|  | PPT leg (ln) | 159 |  |  |  |  |
|  | CPM Lumbar | 138 | 0.12 | 0.06 | 0.52 | 0.56 |
|  | CPM Leg | 134 |  |  |  |  |
| Fatigue | Resting Fatigue (NRS) | 171 | 0.14 | 0.29 | 0.54 | 0.53 |
|  | **Movement Fatigue (6MWT)** | 160 | **0.005** | **0.006** | 0.75 | 0.81 |
|  | Movement Fatigue (5TSTS) | 160 | 0.93 | 0.93 | 0.74 | 0.76 |
|  | Multidimensional (MAF) | 159 | 0.04 | 0.04 | 0.72 | 0.77 |
| Psychological constructs | Catastrophizing (PCS) | 160 | 0.13 | 0.12 | 0.37 | 0.47 |
|  | Fear of movement (TSK) | 160 | 0.38 | 0.35 | 0.16 | 0.19 |
| Disease impact | FIQR Total | 171 | 0.02 | 0.03 | 0.80 | 0.92 |
| Quality of life | **Physical (SF-36 PCS)** | 158 | **<0.001** | **0.005** | **0.001** | **0.003** |
|  | Emotional (SF-36 MCS) | 158 | 0.95 | 0.99 | 0.31 | 0.32 |

†Repeated measures ANOVA, repeated across locations, no interaction between group and location.

**Table S3. Correlation coefficients between function variables.**

| Function | 6MWT (ft) | 5TSTS (sec) [ln] | PROMIS- PF |
| --- | --- | --- | --- |
| 5TSTS (sec) [ln] | **-0.57**** | - |  |
| PROMIS- PF | **0.45**** | **-0.42**** | - |
| SF-36 PF | **0.46**** | **-0.35**** | **0.78**** |

5TSTS = five times sit-to-stand; PROMIS-PF = Patient reported outcomes – physical function scale; SF-36 PF = short form 36 physical function scale.

* p ≤ 0.01; ** P ≤ 0.001

**Table S4. Correlation coefficients between pain variables.**

| Pain | Resting (0-10) | Movement (6MWT) | Movement (5TSTS) | BPI Severity |
| --- | --- | --- | --- | --- |
| Movement (6MWT) | **0.69**** | - |  |  |
| Movement (5TSTS) | **0.49**** | **0.54**** | - |  |
| BPI severity | **0.80**** | **0.67**** | **0.50**** | - |
| BPI Interference | **0.49**** | **0.45**** | **0.40**** | **0.53**** |

6MWT = six-minute walk test; 5TSTS = five times sit-to-stand; BPI = Brief pain inventory.

* p ≤ 0.01; ** P ≤ 0.001

**Table S5. Correlation coefficients between pain sensitivity variables.**

| Pain Sensitivity | PPT cervical [ln] | PPT lumbar [ln] | PPT leg [ln] | CPM lumbar (%) |
| --- | --- | --- | --- | --- |
| PPT lumbar (kPa) [ln] | **0.81**** | - |  |  |
| PPT leg (kPa) [ln] | **0.65**** | **0.72**** | - |  |
| CPM lumbar (%) | -0.11 | -0.05 | -0.03 | - |
| CPM leg (%) | -0.20 | -0.02 | -0.04 | **0.51**** |

PPT= pressure pain threshold; CPM = conditioned pain modulation.

* p ≤ 0.01; ** P ≤ 0.001

**Table S6. Correlation coefficients between fatigue variables.**

| Fatigue | Resting (0-10) | Movement (6MWT) | Movement (5TSTS) |
| --- | --- | --- | --- |
| Movement (6MWT) | **0.55**** | - |  |
| Movement (5TSTS) | **0.44**** | **0.63**** | - |
| MAF | **0.53**** | **0.44**** | **0.43**** |

6MWT = six-minute walk test; 5TSTS = five times sit-to-stand; MAF = Multidimensional Assessment of Fatigue scale.

* p ≤ 0.01; ** P ≤ 0.001

**Table S7. Correlation coefficients between psychological constructs, disease impact, and QoL variables.**

|  | PCS | TSK | FIQ-R | SF-36 PCS |
| --- | --- | --- | --- | --- |
| TSK | **0.49**** |  |  |  |
| FIQ-R | **0.48**** | **0.35**** |  |  |
| SF-36 PCS | -0.15 | **-0.27**** | **-0.52**** |  |
| SF-36 MCS | **-0.55**** | **-0.25*** | **-0.51**** | -0.14 |

TSK = Tampa Scale of Kinesiophobia; PCS = Pain Catastrophizing Scale; FIQ-R = Fibromyalgia Impact Questionnaire - Revised; SF-36 PCS = Short Form 36 - Physical quality of life scale; SF-36 MCS = Short Form 36 - Emotional quality of life scale.

* p ≤ 0.01; ** P ≤ 0.001
